# Breast cancer and ATM mutations: treatment implications

**DOI:** 10.1186/s13053-024-00300-9

**Published:** 2024-11-14

**Authors:** Marta Seca, Steven A. Narod

**Affiliations:** 1 Department of Medicine and Surgery, University Milan-Bicocca, Piazza dell ’Ateneo Nuovo, Milan, Italy; 2 Women’s College Research Institute, Women’s College Hospital, 76 Grenville Street, Toronto, ON M5S 1B2 Canada; 3 Dalla Lana School of Public Health, University of Toronto, Toronto, ON Canada

**Keywords:** ATM mutation, Breast cancer, Ovarian cancer

## Abstract

Genetic testing for breast cancer predisposing genes has expanded beyond *BRCA1* and *BRCA2* and now includes panels of 20 or more genes. It is now recommended that all women diagnosed with breast cancer at age 65 or below be offered testing for an extended gene panel. The rationale for testing includes personalizing the management of breast cancer according to the mutation found. For *BRCA1* and *BRCA2* carriers, the finding of a mutation has clear implications for cancer management, but for other genes, such as *ATM*, the management implications are less clear. Women with an *ATM* mutation have a lifetime risk of breast cancer of approximately 25%, the majority of which are ER-positive. The risk of ovarian cancer is approximately 5%. It is not yet clear how the identification of an *ATM* mutation in a patient newly diagnosed with breast cancer should impact on her treatment and follow-up. At present, these women are treated in the same way as women without a mutation. It is important that large prospective studies be conducted looking at various treatment modalities in women with breast cancer and an *ATM* mutation in order to optimize outcomes.

## Background

It has long been known that female carriers of an *ATM* mutation are at increased risk of breast cancer [1]. This conclusion was based on the observation that the mothers of children with ataxia-telangiectasia (AT) are at increased risk of breast cancer. In 1995, the *ATM* gene was discovered and was found to be responsible for ataxia–telangiectasia. *ATM* is a protein kinase whose activity is enhanced by DNA damage. The *ATM* gene is crucial for repairing double-strand DNA breaks. Homozygous mutations in *ATM* lead to ataxia-telangiectasia, a rare autosomal recessive condition characterized by cerebellar degeneration, oculocutaneous telangiectasia, and immunodeficiency [2, 3].

The *BRCA1* gene was identified in 1994 and the *BRCA2* gene was identified in 1995 [4]. Since genetic testing for breast cancer susceptibility was introduced in 1995, eligibility criteria for testing have widened considerably, and now, women diagnosed with breast cancer before age 65 are eligible for testing. In addition, the panel of genes to be tested has expanded to 20 or more. The *ATM* gene, which is responsible for AT, is included in all of the panels, despite no clear recommendations for the care of women found to carry a mutation. Other moderate penetrance genes of importance include *PALB2* and *CHEK2*. For most of these genes the clinical implications are uncertain. In the current paper, we review the risks of breast and ovarian cancer (penetrance) and discuss the management of breast cancers that occur in carriers of an *ATM* mutation.

## Historical background

### Family studies

Given the recessive nature of AT is assumed that both parents of children with AT are heterozygous carriers of an *ATM* mutation. Early studies reviewed the cancer family histories of children with AT. Based on this premise, Swift et al. looked for breast cancer in 10,211 blood relatives of AT patients [5]. They estimated that women with heterozygous *ATM* mutations had a 5.1-fold increased risk of breast cancer, compared to the general population (*P* = 0.009). In a later study, Thompson et al. [6], identified 118 cases of breast cancer in 1,160 relatives of AT patients. The relative risk of breast cancer in carriers was estimated to be 2.23 (95% CI: 1.16 to 4.28) compared with the general population but was 4.94 (95% CI = 1.90 to 12.9) for women younger than age 50.

### Case control studies

Several case-control studies have been done to estimate the odd ratio for developing breast cancer for *ATM* mutation carriers. They compare the mutation prevalence in affected women and unaffected population-based controls. By using the odds ratio thereby generated, they estimate the penetrance by multiplying the population risk and the odds ratio.

In a review of seven studies conducted in United States, the United Kingdom, France, or Scandinavia, van Os et al. [7]. reported that women with pathogenic *ATM* variants have a relative risk of 3.0 (90% CI 2.1–4.5 *P* < 0.0001) of developing breast cancer, compared to the general population.

A recent study by the Breast Cancer Consortium found an *ATM* mutation in 294 of 60,466 women with breast cancer (0.49%) and 150 or 53,461 controls (0.28%) (OR 2.10, 95% CI 1.71–2.57). *ATM* was more strongly associated with ER-positive cancer (OR = 2.33) than with ER-negative cancer (OR = 1.01) [8].

In a second large study (CARRIERS study) the prevalence of *ATM* mutations came from sequencing large number of unselected breast cancer patients and controls [9]. In the CARRIERS study an *ATM* mutation was found in 253 of 32,247 breast cancer patients (0.78%) and in 134 of 32,544 controls (0.41%). The odds ratio was 1.96 for ER-positive breast cancer and 1.04 for ER-negative breast cancer.

### Prospective studies

Prospective studies follow unaffected *ATM* carriers forward in time to estimate the incidence of breast cancer in *ATM* carriers. The latter studies are more accurate but are considerably more difficult to conduct and take a long period of time. To my knowledge no prospective studies of healthy *ATM* carriers have been conducted.

In a summary of family-based studies and case-control studies, Marabella et al. [10], et al. estimated that female carriers of *ATM* variants face a risk of breast cancer of 6.0% to age 50 and 33% to age 80. These findings suggest that women with *ATM* mutations face a risk of developing breast cancer that is two to three times higher than the general public and the majority of these are ER-positive. This is equivalent to a lifetime risk of roughly 25%. The risk may vary by specific mutation (see below).

#### Interpretation of mutations

In the BCAC study the prevalence of mutations in breast cancer patients was 0.5% and, in the CARRIERS, study the prevalence was 0.8% [8, 9]. Currently, *ATM* mutations are classified according to standard nomenclature as benign, likely benign, variants of unknow significance (VUS), likely pathogenic and pathogenic. Physicians are directed to consult the Clinvar database for the interpretation of individual mutations. *ATM* mutations are more difficult to interpret than mutations in other breast cancer susceptibility genes because the risk of cancer may vary by mutation and many of the high penetrance mutations are missense mutations, which are difficult to distinguish from benign polymorphisms. *ATM* mutations may or may not be protein-truncating. It appears that non-truncating mutations may be associated with higher risks for breast cancer than non-truncating mutations, but there is little data on the risks associated with individual mutations. One common missense mutation c7271T > G has been associated with a high risk of invasive ductal breast cancer (OR = 3.8). There is also a Finnish founder mutation (c7579G > C) which is reported to have a higher than average penetrance [11].

Variants of unknown significance (VUS) comprise approximately one-half of *ATM* variants recorded. At this point we do not recommend any clinical actions be taken for breast cancer patients with a VUS and we do not recommend that relatives be tested for this type of mutation.

*ATM* mutations may also be classified as ‘likely deleterious’. The clinical consequences of these are difficult to interpret. The ‘*Genesis study’* compared protein-truncating variants with missense variants and concluded that that those women with truncating mutations had a higher risk of breast cancer than those with ‘likely deleterious’ missense variants [12]. However, for some missense variants, such as c7271T > G, the risk of cancer is predicted to be higher than that of truncating variants.

### ATM and contralateral breast Cancer

Studies to date do not show a significantly increased risk of contralateral breast cancer for women with breast cancer and at *ATM* mutation. In a large study, Yadav et al. [13]. reported that carriers of *ATM* pathogenic variants did not show a significantly increased risk of contralateral breast cancer compared to non-carriers (HR 1.2, 95% CI 0.6–2.6, *p* = 0.56. Overall, these results argue against recommending contralateral prophylactic mastectomy for reducing contralateral breast cancer in *ATM* pathogenic variant carriers.

### ATM and other cancers

*ATM* has been associated with a range of cancers in addition to breast cancer. These include pancreatic, prostate, gastric, melanoma, colon and ovarian [2]. For the purpose of this review, we do not recommend screening for pancreatic and gastric cancer as the cancer risks are not that high at screening is not simple. Of particular interest is the risk of ovarian cancer as this can be prevented by oophorectomy and is used in the treatment of high risk, early-onset breast cancer [14, 15]. Oophorectomy has been shown to diminish breast cancer mortality in carriers of *BRCA1*, *BRCA2* and *CHEK2* mutation mutations but has not yet been studied in breast cancer patients with *ATM* mutations. In a recent review of ovarian suppression by the Early Breast Cancer Trialists Collaborative Group, ovarian suppression was shown to be beneficial in terms of reducing breast cancer mortality in young women with ER-positive breast cancer [16]. The majority of breast cancers in carriers of *ATM* mutations are ER-positive.

Several researchers have investigated the role of *ATM* mutations in the development of ovarian cancer [17]. Lilyquist et al. [18], studied frequency of pathogenic ATM alterations in 7,768 ovarian cancer cases referred to a single clinical laboratory. The authors reported a standard standardized risk ratio (SRR) for ovarian cancer of 2.25 (95% 1.7– 2.9) compared to mutation frequencies in the Exome Association cancer dataset. In 2016, Norquist et al. [19]. studied 1,915 women with ovarian cancer from the University of Washington Medical Center and two sites of the Gynecology Oncology Group (GOG) in the United States. They compared mutation frequencies in ovarian cancer cases to those in the NHLBI Exome Sequencing Project (ESP) and the ExAC. Their findings revealed a 2.4 risk of ovarian cancer associated with ATM mutations (OR = 2.4, 95% CI 1.2–4.4, *p* = 0.01) in the ExAC cohort. In 2017, Kurian et al. [20]. analyzed a cohort of 95,561 women who underwent extended next-generation sequencing (NGS) for hereditary cancer risk assessment in the US. *ATM* mutations were linked to at least a small increased risk of ovarian cancer (OR 1.69, 95% CI 1.19–2.4, *p* = 0.0032). Similarly, in a 2018 case-control study Lu et al. [21] examined 2,051 women diagnosed with ovarian cancer in United States. They reported that *ATM* mutations as pathogenic variants were associated with three-fold increased risk of ovarian cancer (OR 2.85; 95% CI 1.30–6.32).

Overall, these studies suggest that *ATM* mutation led to a modest increase in risk of ovarian cancer. Based on the relative risks quoted above this is equivalent to a lifetime risk of ovarian cancer of about three to five%. This level of risk suggests that it is reasonable perform a preventive oophorectomy after menopause in *ATM* carriers without breast cancer. For those with breast cancer, there may be a benefit of oophorectomy breast cancer mortality, but this has not yet been verified. This is an important question to study going forward.

### Management considerations

There is limited information in the literature regarding the implications of carrying and *ATM* mutation on the therapeutic approach. For women with pathogenic *BRCA* variants, PARP inhibitors have proven to be an effective treatment option for both breast and ovarian cancer [22]. Since *ATM* is involved in the same DNA repair pathway, there is growing interest in whether these inhibitors could also be a safe and effective treatment for patients with *ATM* mutations [23].

In their latest update, the National Comprehensive Cancer Network (NCCN) guidelines, although suggesting a strong association between high-risk ATM mutations (c.7271T > G) and both breast and ovarian cancer, do not recommend any risk-reducing surgery [24].

#### Radiotherapy

AT is characterized by radiosensitivity. For this reason, it is important to know if radiotherapy as used for the treatment of breast cancer post-surgery is beneficial or poses hazards to women who carry an *ATM* mutation.

In a 2020 case-control study, Reiner et al. [25]. investigated the impact of radiation treatment (RT) on the risk of contralateral breast cancer (CBC) in women previously treated for breast cancer and carrying pathogenic variants in ATM or other genes. Their analysis, which included 708 cases of women with contralateral breast cancer and 1,399 controls with unilateral breast cancer, found that carriers of *ATM* pathogenic variants showed a small but not statistically significant increase in contralateral breast cancer risk (RR = 1.68, 95% CI = 0.75–3.76, *p* = 0.20). The authors reported that women carrying missense variants of uncertain significance (VUS) in *ATM* had an elevated risk of CBC contralateral breast cancer, particularly among those who underwent RT (RR = 2.98, 95% CI = 1.31–6.80). On the whole studies to date do not suggest that women with breast cancer and an *ATM* mutation should avoid radiotherapy or to reduce the dose.

On the other hand, radiotherapy has always been a hallmark of breast cancer treatment; but there has been considerable discussion regarding its safety in patients with ATM mutations, given their known increased radiosensitivity [2, 26, 27]. In 2017, the NCCN recommended not to avoid radiotherapy in this subgroup but to consider it carefully. Recently, Bensenane et al. [28] conducted an observational retrospective study, which demonstrated no significant acute or late toxicities following breast radiation therapy among patients carrying a heterozygous rare variant of the *ATM* gene. This suggests that with careful consideration, radiotherapy may still be a viable treatment option for this higher risk population.

### Going forward

At present women with breast cancer diagnosed under age 65 are recommended to have genetic testing for *ATM* mutations as part of a comprehensive panel. There is no evidence that this test is of any benefit to the patient as there are no specific recommendations for treatment. The association of *ATM* mutations is restricted to those with ER-positive breast cancer and given the lack of association between *ATM* and ER-negative breast cancer it is safe to assume that ER-negative cancer that occur in AT carriers are examples of sporadic cancer and are probably not manifestation of an underling susceptibility and should be treated according to conventional means.

For women with ER-positive cancer there is little evidence on the effect of ovarian suppression, hormonal therapies or chemotherapy and these are considerations for future studies. The risk of ovarian cancer is approximately three to five% and this may offer some justification to consider surgical ovarian suppression, in particular for young women with high-risk cancers. In the lack of an increased risk of contralateral breast cancer, bilateral mastectomy is not recommended but maybe pursued at the choice of the patient. There is no contraindication to radiotherapy to the breast, lymph nodes or chest wall. Future studies should also consider various chemotherapy regiments, in the neoadjuvant and adjuvant setting to see the relative effectiveness in preventing distant recurrence and mortality in women with breast cancer and an *ATM* mutation.

## Data Availability

No datasets were generated or analysed during the current study.
